# Caregiver-Related Factors Associated with HIV Status Disclosure and Treatment-Related Indicators Among Children and Adolescents Receiving ART in Rural Eastern Cape, South Africa

**DOI:** 10.3390/tropicalmed11100275

**Published:** 2026-09-30

**Authors:** Siwongiwe Qwase, Laston Gonah, Guillermo Alfredo Pulido Estrada, Ziphelele Peter, Teke R. Apalata, Monwabisi Faleni

**Affiliations:** 1 School of Public Health, Faculty of Medicine and Health Sciences, Walter Sisulu University, Mthatha 5100, South Africa; swoshiey@gmail.com (S.Q.); lgonah@wsu.ac.za (L.G.); gpulidoestrada@wsu.ac.za (G.A.P.E.); zpeter@wsu.ac.za (Z.P.); 2 WSU Society and Health Research Institute, Walter Sisulu University, Mthatha 5100, South Africa; 3 School of Pathology, Faculty of Medicine and Health Sciences, Walter Sisulu University, Mthatha 5100, South Africa; tapalata@wsu.ac.za

**Keywords:** HIV disclosure, adolescents, antiretroviral therapy, adherence, viral suppression, caregiver readiness, paediatric HIV

## Abstract

Introduction: HIV status disclosure to children remains complex and constrained by child-, caregiver-, and health-system-level factors. Aim: To assess factors associated with caregiver-reported HIV status disclosure among children and adolescents receiving antiretroviral therapy (ART) and explore associations between disclosure status and selected treatment-related indicators in rural Eastern Cape province. Methods: A cross-sectional study of 87 caregiver–child dyads was conducted across five public ART clinics. Data were collected using caregiver questionnaires and abstraction from ART records. Bivariate associations were assessed using Pearson’s chi-square or Fisher’s exact tests, and multivariable binary logistic regression was performed to identify caregiver-related factors independently associated with HIV status non-disclosure. Results: Overall, 44.8% (*n* = 39) of children and adolescents had been informed of their HIV status. Status disclosure was associated with older age and legal guardian caregiving (*p* < 0.001, respectively). Participants who had been disclosed to had higher rates of viral suppression (74.4% vs. 29.2%), optimal ART adherence (87.2% vs. 45.8%), and independent medication administration (82.1% vs. 33.3%) (all *p* < 0.001). In multivariable analysis, caregiver readiness to discuss HIV was associated with lower odds of non-disclosure (AOR = 0.037; 95% CI: 0.004–0.381), whereas the perceived need for additional disclosure training and counselling (AOR = 15.97; 95% CI: 3.05–83.53) and biological-parent caregiving (AOR = 11.92; 95% CI: 2.16–65.67) were associated with higher odds of non-disclosure. Conclusion: HIV disclosure was associated with developmental, caregiving, and treatment-related factors. Strengthening structured caregiver preparation and disclosure support within routine paediatric HIV services may improve implementation, although longitudinal studies are needed to clarify temporal relationships and causal pathways.

## 1. Introduction

Global antiretroviral therapy (ART) rollout has transformed the prognosis of perinatally acquired HIV from a rapidly fatal paediatric illness into a manageable chronic condition, enabling more children to survive into adolescence and adulthood [1,2]. As children live and grow with the virus, long-term treatment success depends not only on sustained access to medication but also on gradual engagement in self-management and informed participation in care. Central to this transition is HIV status disclosure by the caregiver; the process through which children are informed about their own HIV diagnosis [2,3,4].

International guidelines, including recommendations from the World Health Organization (WHO) and national HIV programmes in high-burden settings, recommend gradual and developmentally appropriate HIV disclosure as a component of quality paediatric HIV care [3,4]. WHO conceptualises disclosure as a staged communication process that aligns with developmental readiness, psychosocial support, and caregiver involvement, beginning with foundational health concepts and progressing toward full disclosure by late childhood to support treatment engagement and autonomy [4]. Ethically, status disclosure supports children’s evolving autonomy and health-related decision-making. Psychologically, it may reduce confusion surrounding long-term care and repeated healthcare encounters while strengthening communication between children and caregivers. Clinically, disclosure has been proposed as a key factor that may support long-term treatment adherence and engagement [3,4,5,6].

Despite these recommendations and potential benefits, HIV status disclosure remains delayed and inconsistently implemented, particularly in sub-Saharan Africa, which bears the greatest global burden of paediatric HIV [7,8]. Status disclosure rates across the region remain variable and often fall below guideline expectations, with evidence suggesting that delays may result from complex and interacting influences operating across child, caregiver, community, and health-system levels [6,9,10]. Among the multiple factors that may influence HIV status disclosure, caregiver-related factors have received increasing attention in the literature [3,4,8,11]. Reported caregiver concerns include fear of causing emotional distress, anticipated stigma, uncertainty regarding children’s readiness to understand HIV, and limited confidence in initiating disclosure discussions [9,12,13]. These concerns may vary across caregiving contexts and may be compounded by caregiver capacity and health-system support within routine HIV services [14].

In addition to factors influencing disclosure practices, growing attention has focused on whether HIV disclosure status is associated with broader aspects of treatment engagement among children and adolescents receiving ART [8,14]. Existing studies have reported inconsistent findings regarding associations between disclosure and indicators such as adherence behaviour and viral suppression, partly due to differences in study populations, study designs, and approaches to measuring disclosure [8,10,14,15,16]. These variations highlight the need for context-specific evidence to better understand status disclosure patterns and their relationship with treatment-related indicators within routine paediatric HIV care.

Against this background, this study assessed factors associated with caregiver-reported HIV status disclosure and examined associations between disclosure and selected treatment-related indicators among children and adolescents receiving ART in a rural South African setting. By generating context-specific evidence from routine paediatric HIV services, the study seeks to inform disclosure-support approaches and priorities for future research.

## 2. Materials and Methods

### 2.1. Study Design and Setting

This facility-based, cross-sectional study was conducted from July to September 2025 across five public-sector ART clinics in the rural Engcobo sub-district of Chris Hani District, Eastern Cape. These facilities were selected because they provide routine paediatric and adolescent HIV services and collectively serve geographically dispersed communities with limited access to specialised HIV support services. The study setting reflects a predominantly rural, resource-limited public healthcare context characterised by socioeconomic vulnerability and dependence on public-sector HIV programmes. Eastern Cape is among South African provinces with a high HIV burden, with paediatric HIV care increasingly focused on sustaining treatment engagement and transition into adolescent self-management [15].

### 2.2. Study Population and Sampling

The sample size for this study was estimated using Cochran’s formula based on an assumed viral suppression prevalence of 80%, a 95% confidence level, and a 10% margin of error. After allowing for a 10% non-response or invalid response rate and considering the anticipated number of eligible paediatric ART attendees across participating facilities during the study period, the target sample size was 86 caregiver–child dyads.

The study population comprised children and adolescents living with HIV and their primary caregivers, recruited as caregiver–child dyads (1:1 pairing) during routine clinic attendance. Eligible participants were children aged 6–19 years with confirmed perinatally acquired HIV infection documented in clinic ART records, who had received ART for at least 12 months prior to enrolment and attended the clinic accompanied by a consenting primary caregiver. Primary caregivers were defined as biological parents or legally recognised guardians with routine responsibility for medication administration and clinic attendance. Children were excluded if they had severe cognitive impairment that could prevent meaningful participation, or acute illness requiring emergency care. Where multiple caregivers were involved in the child’s care, the caregiver with primary responsibility for daily HIV management and treatment oversight was enrolled. Where siblings attended the clinic simultaneously, each eligible caregiver–child dyad was enrolled independently, provided eligibility criteria were met.

A consecutive sampling approach was used, whereby all eligible caregiver–child dyads attending participating facilities during the study period were invited to participate until the target sample size was reached. This pragmatic recruitment strategy best suited the study setting and participant availability, though it may have limited statistical precision and generalisability. Participant recruitment was conducted during routine clinic visits, during which clinic records were verified by trained study personnel in collaboration with clinic staff to determine eligibility. Eligible caregiver–child dyads were approached privately following routine clinical services, provided with study information, and invited to participate. No incentives were offered.

### 2.3. Data Collection and Measures

Data were collected using a structured, interviewer-administered caregiver questionnaire, complemented by clinical data abstracted from ART records. The primary outcome was caregiver-reported HIV status disclosure to the child, measured as a binary variable (yes/no) reflecting caregiver report of whether the child had been informed of their HIV diagnosis. This measure captured disclosure status without distinguishing between partial and full disclosure, timing, quality, child comprehension, healthcare worker involvement, or post-disclosure counselling.

Independent variables included child- and caregiver-related factors. Child-related variables comprised age, sex, viral load suppression, ART adherence, and medication administration responsibility. Viral load suppression was defined as the most recent clinic-documented viral load result and categorised as suppressed (≤1000 copies/mL) or non-suppressed (>1000 copies/mL). Since serial viral load measurements were not analysed, this variable represented point-in-time rather than sustained suppression. ART adherence was assessed through the caregiver’s self-report of the child’s medication-taking behaviour over the preceding 30 days and categorised as always, sometimes, or rarely adherent. These reports were reviewed alongside clinic retention records for contextual interpretation only and were not incorporated into a composite adherence measure. For interpretive purposes, optimal adherence referred to participants classified as always adherent. This adherence measure relied on caregiver report only and did not incorporate validated adherence instruments, pharmacy refill data, pill counts, or electronic monitoring.

Caregiver-related variables included self-reported readiness to discuss HIV with the child, belief that the child would understand HIV, receipt of emotional support, knowledge of other caregivers who had disclosed HIV status, perceived need for additional disclosure training or counselling, fear of the child’s reaction to disclosure, disclosure support preferences (counselling, support groups, or step-by-step guidance), and caregiver–child relationship. Missing data were minimal, with no variable exceeding 5% missingness, and were handled using complete-case analysis.

### 2.4. Data Analysis

Statistical analyses were performed using Statistical Package for the Social Sciences (SPSS) Statistics for Windows, Version 30.0 (IBM Corp., Armonk, NY, USA).. Descriptive statistics were used to summarise participant characteristics using frequencies and percentages. Bivariate associations between HIV status disclosure and independent variables were assessed using Pearson’s chi-square test or Fisher’s exact test when >20% of expected cell counts were <5. Variables included in the multivariable model were selected based on conceptual relevance [3,4,14,15,16] and supported by bivariate findings to minimise overfitting and sparse-data instability. Because viral suppression, adherence, medication administration responsibility, and adherence support could plausibly occur after disclosure and therefore may lie along the pathway between disclosure and treatment engagement, these variables were examined descriptively and were not included in the multivariable model to avoid adjustment for potential mediators.

A binary logistic regression model using the Enter method [17] was fitted with four caregiver-related predictors: caregiver readiness to discuss HIV, perceived need for additional disclosure training/counselling, knowledge of other caregivers who had disclosed HIV status, and caregiver–child relationship. Model fit was assessed using the Hosmer–Lemeshow goodness-of-fit test and Nagelkerke’s R^2^. Adjusted odds ratios with 95% confidence intervals (CIs) were reported, and statistical significance was defined as *p* < 0.05. Given the limited sample size and outcome events, multivariable estimates may have been affected by sparse-data bias and should therefore be interpreted as exploratory. Given the modest sample size and sparse cells for some predictor categories, the multivariable analysis was considered exploratory and interpreted cautiously, with emphasis placed on the direction and consistency of associations rather than the precision of individual estimates.

As a sensitivity analysis, the multivariable logistic regression model was additionally adjusted for child age group to assess whether the observed caregiver-related associations were robust after accounting for this potential confounder.

### 2.5. Ethical Considerations

Ethical approval was obtained from the Walter Sisulu University Faculty of Medicine and Health Sciences Research Ethics Committee (REF: WSU HREC 161/2025) and the Eastern Cape Department of Health (EC_202507_034). Written informed consent was obtained from all participating caregivers, while written assent was obtained from children aged ≥12 years, and younger children received age-appropriate study information.

Participation in the study did not involve or trigger HIV status disclosure among children who were previously unaware of their diagnosis. To protect confidentiality and minimise disclosure-related distress, interviews were conducted in private settings, and participants requiring psychosocial support were referred to facility-linked counselling services. All study data were anonymised and stored securely.

## 3. Results

### 3.1. Sociodemographic Characteristics

A total of 87 caregiver–child dyads were included in the final analysis. Participant sociodemographic characteristics are summarised in Table 1. The sample was relatively balanced by sex, comprising 52.9% females (*n* = 46) and 47.1% males (*n* = 41). Most children and adolescents were aged 10–13 years (54.0%, *n* = 47), while children aged 6–9 years accounted for 18.4% (*n* = 16). Most participants were cared for by biological parents (65.5%, *n* = 57), whereas 34.5% (*n* = 30) were under the care of legal guardians. Additional caregiver-related and treatment-related variables are presented in subsequent analyses.

### 3.2. Prevalence of HIV Status Disclosure

Overall, 44.8% (*n* = 39) of children and adolescents had reportedly been informed of their HIV status, whereas 55.2% (*n* = 48) had not yet been informed. Disclosure prevalence was based on caregiver report and therefore reflects the caregiver’s report of whether the child had been informed of their HIV diagnosis.

### 3.3. Factors Associated with HIV Status Disclosure

Participant characteristics associated with HIV status disclosure are presented in Table 2. Child sex was not significantly associated with disclosure status (OR = 1.35; 95% CI: 0.58–3.16; *p* = 0.484); however, child age was strongly associated with disclosure. Compared with children aged 6–9 years, those aged 10–13 years had 3.61 times higher odds of disclosure (OR = 3.61; 95% CI: 0.73–17.88), although this association was not statistically significant, whereas children aged 14–19 years had significantly higher odds of disclosure (OR = 49.00; 95% CI: 7.24–331.60; *p* < 0.001). Caregiving arrangement was also significantly associated with disclosure status. Children cared for by legal guardians had 8.42 times higher odds of having been informed of their HIV status than those cared for by biological parents (OR = 8.42; 95% CI: 3.03–23.45; *p* < 0.001).

Caregiver-related factors were significantly associated with HIV status disclosure (Table 3). Caregivers who reported being ready to discuss HIV with their child had substantially higher odds of disclosure than those who were not ready (OR = 76.00; 95% CI: 9.22–626.74; *p* < 0.001). Similarly, caregivers who believed that their child would understand HIV had significantly higher odds of disclosure compared with those who did not hold this belief (OR = 4.38; 95% CI: 1.30–14.73; *p* = 0.011).

Knowledge of other caregivers who had previously disclosed HIV status was also associated with disclosure, with these caregivers demonstrating 4.56 times higher odds of disclosure than those without such knowledge (OR = 4.56; 95% CI: 1.76–11.81; *p* = 0.001). Again, caregivers who reported receiving emotional support were more likely to report disclosure than those who did not (OR = 3.11; 95% CI: 1.05–9.21; *p* = 0.036). In contrast, caregivers who reported needing additional training or counselling on HIV disclosure had significantly lower odds of having disclosed their child’s HIV status than those who did not report such needs (OR = 0.03; 95% CI: 0.009–0.11; *p* < 0.001). Fear of how the child would react was also significantly associated with disclosure status (*p* = 0.011); however, a crude odds ratio could not be reliably estimated because one category contained no non-disclosed participants. Disclosure support preferences differed significantly between the disclosure groups (*p* = 0.016). Caregivers of disclosed to children more frequently identified support groups as a preferred facilitator of disclosure, whereas caregivers of non-disclosed to children more commonly preferred step-by-step guidance or counselling.

### 3.4. Disclosure Status and Treatment-Related Indicators

Treatment-related indicators associated with HIV status disclosure are presented in Table 4. Children and adolescents with viral load suppression had significantly higher odds of disclosure than those without viral load suppression (OR = 7.04; 95% CI: 2.72–18.23; *p* < 0.001). Similarly, participants classified as always adherent had higher odds of disclosure than those who were rarely adherent (OR = 6.95; 95% CI: 1.37–35.24), whereas those who were sometimes adherent did not differ significantly from the reference group (OR = 0.79; 95% CI: 0.11–5.65; *p* < 0.001 for the overall association). Medication administration was also significantly associated with disclosure status. Children who self-administered their medication had 9.14 times higher odds of disclosure than those whose medication was administered by a parent (OR = 9.14; 95% CI: 3.32–25.20; *p* < 0.001). In addition, participants with adherence support systems had significantly higher odds of disclosure than those without such support systems (OR = 11.04; 95% CI: 2.99–40.81; *p* < 0.001).

### 3.5. Multivariable Predictors of HIV Status Non-Disclosure

Multivariable logistic regression analysis identified several caregiver-related factors independently associated with HIV status non-disclosure (Table 5). The regression outcome was coded as non-disclosure; therefore, adjusted odds ratios (AORs) greater than one indicate higher odds of remaining undisclosed, whereas AORs less than one indicate lower odds. Caregivers who reported being ready to discuss HIV with their child had significantly lower odds of non-disclosure than those who were not ready (AOR = 0.037; 95% CI: 0.004–0.381; *p* = 0.006). Conversely, children cared for by biological parents had significantly higher odds of remaining undisclosed than those cared for by legal guardians (AOR = 11.92; 95% CI: 2.16–65.67; *p* = 0.004).

Caregivers who expressed a need for additional training or counselling on HIV disclosure had approximately 16 times higher odds of non-disclosure than those who did not report such needs (AOR = 15.97; 95% CI: 3.05–83.53; *p* = 0.001). Knowledge of other caregivers who had previously disclosed HIV status was associated with lower odds of non-disclosure; however, this association was not statistically significant after adjustment for the other variables in the model (AOR = 0.339; 95% CI: 0.071–1.612; *p* = 0.174).

The overall logistic regression model was statistically significant (Omnibus χ^2^ = 73.439, df = 4, *p* < 0.001) and demonstrated good fit to the data, as indicated by a non-significant Hosmer–Lemeshow goodness-of-fit test (χ^2^ = 5.097, df = 6, *p* = 0.531). The model explained approximately 76.3% of the variation in HIV status non-disclosure (Nagelkerke R^2^ = 0.763) and correctly classified 88.5% of participants. Although the model demonstrated satisfactory fit, the relatively small sample size and wide confidence intervals for some estimates suggest that the findings should be interpreted as exploratory and warrant confirmation in larger studies.

An additional multivariable logistic regression model adjusting for child age group was performed to assess the robustness of the caregiver-related associations. Adjustment for child age did not materially alter the associations between caregiver readiness to discuss HIV and the perceived need for additional training or counselling and HIV status non-disclosure, both of which remained statistically significant. However, the association between caregiver–child relationship and non-disclosure was attenuated and was no longer statistically significant after adjustment for age (AOR = 6.51; 95% CI: 0.85–50.09; *p* = 0.072), suggesting that the observed association may have been partially explained by differences in child age. Overall, these findings indicate that caregiver readiness and perceived need for additional training remained statistically significant after age adjustment, whereas the association with caregiver relationship was attenuated.

## 4. Discussion

This study examined caregiver-reported HIV status disclosure among children and adolescents receiving ART in rural South Africa and assessed its associations with treatment-related indicators and caregiver characteristics. Three main findings emerged: prevalence of HIV status disclosure to children was lower than non-disclosure; disclosure was more common among older children and those cared for by legal guardians; and disclosure clustered with favourable treatment indicators while remaining independently associated with caregiver readiness and disclosure support needs. These findings suggest that paediatric HIV status disclosure extends beyond a single communication event and reflects a broader developmental and caregiving process shaped by developmental readiness, caregiver capacity, and engagement with HIV care over time.

The low prevalence of disclosure is consistent with findings across sub-Saharan Africa showing persistent delays despite expanded ART access and established disclosure guidelines [7,10,11,14,16]. This consistency suggests that the availability of treatment alone does not ensure timely disclosure and highlights the challenges of implementing disclosure within routine paediatric HIV care [1,4]. Importantly, delayed disclosure should not be interpreted as caregiver reluctance or inadequate caregiving, but rather as a decision occurring within complex emotional, social, and health-system contexts. This broader context may also help explain why disclosure in this study occurred predominantly later in childhood and differed according to caregiving arrangement.

Status disclosure was associated with age, with disclosure occurring predominantly among older age groups. This pattern aligns with existing evidence indicating that disclosure is often postponed until children demonstrate greater cognitive maturity, assume responsibility for treatment, question long-term medication use, or approach transition to adolescent care [3,4,13,14]. Although postponement may reflect efforts to protect children from emotional distress, delayed disclosure may limit opportunities for gradual understanding, supported adjustment, and early engagement in treatment self-management [4,14]. These findings support recommendations that frame disclosure as a staged and developmentally appropriate process rather than a single delayed event.

Children cared for by legal guardians also demonstrated higher disclosure prevalence than those cared for by biological parents. However, this association was attenuated after adjustment for child age in a sensitivity analysis, suggesting that differences in the age distribution of children across caregiving groups may partly explain this finding. This difference should not be interpreted as reflecting caregiving quality but may instead indicate variation in the emotional and relational context surrounding disclosure decisions. Biological parents may experience additional barriers, such as guilt, fear of blame, anxiety about discussing HIV acquisition, and concerns about damaging the parent–child relationship, which may contribute to delayed disclosure, whereas legal guardians may encounter fewer of these emotional constraints, potentially facilitating earlier disclosure [7,11,12,13].

Status disclosure was also associated with more favourable treatment-related indicators, including viral load suppression, reported adherence, independent medication administration, and adherence support. However, these findings should be interpreted within the limits of the cross-sectional design, which does not establish temporal sequence or causality. Children with stronger treatment engagement and greater developmental readiness may have been more likely to have been disclosed to, while disclosure itself may support treatment understanding and participation in care [14,16]. These mechanisms are likely interrelated rather than mutually exclusive and suggest that disclosure and treatment engagement may reinforce one another over time rather than operate through a simple causal pathway [14,16]. Interpretation of these findings should consider that status disclosure was measured as a binary caregiver-reported outcome. This binary classification may not fully capture the iterative nature of the disclosure continuum, potentially misclassifying children who received partial health information or masking substantial variations in disclosure timing, depth, comprehension, and post-disclosure support. Accordingly, status disclosure in this study should be interpreted as a broad indicator of disclosure experience rather than a complete representation of the disclosure process.

Multivariable findings further emphasised the importance of caregiver capacity. Caregiver readiness to discuss HIV remained independently associated with lower odds of non-disclosure, whereas reported need for additional training and counselling was associated with higher odds of remaining undisclosed. These findings suggest that disclosure decisions are shaped not only by willingness to disclose but also by caregiver confidence, preparedness, and perceived support [3,4,5,6]. This interpretation is consistent with the broader pattern observed across the study, where caregiver-related factors appeared to play an important role alongside child characteristics in shaping status disclosure.

These findings have implications for paediatric HIV services in sub-Saharan Africa. Disclosure support may require more than the provision of age-based recommendations and should be integrated into routine care through structured caregiver readiness assessment, longitudinal counselling, staged disclosure planning, and post-disclosure follow-up. Targeted support may be particularly valuable for biological parents and caregivers expressing uncertainty or unmet training needs. Embedding disclosure support within existing adherence and retention services may provide a pragmatic approach to strengthening continuity of care across childhood and adolescence.

### Study Limitations

Several limitations should be considered when interpreting these findings. The cross-sectional design precludes establishing temporal relationships or causal inference between disclosure and treatment-related indicators, and reverse causation remains possible. Although the sample was sufficient for exploratory analysis, the limited number of disclosure events may have reduced estimate precision and contributed to sparse-data effects and wide confidence intervals in the multivariable models. Although the logistic regression model converged successfully and demonstrated acceptable goodness of fit, the estimates should be interpreted cautiously because standard maximum-likelihood logistic regression may be sensitive to sparse data in small samples.

Disclosure was measured as a binary caregiver-reported outcome and therefore did not capture important dimensions of the disclosure process, including timing, depth of disclosure, child understanding, healthcare worker involvement, and post-disclosure support. ART adherence was also assessed primarily through caregiver report without validation using objective measures such as pharmacy refill data, introducing the potential for recall and social desirability bias.

In addition, several potentially relevant variables were not available for analysis, including socioeconomic conditions, caregiver education, ART duration, timing of HIV diagnosis, mental health factors, and longitudinal clinical indicators. Although a sensitivity analysis adjusting for child age was performed, the relatively small sample size limited the precision of the age-adjusted estimates, and residual confounding from unmeasured caregiver, household, and health-system characteristics cannot be excluded. Future studies should use longitudinal designs and multidimensional disclosure measures to better characterise disclosure trajectories and their relationship with treatment outcomes.

Despite these limitations, the study provides context-specific evidence on HIV disclosure practices within routine paediatric HIV services and identifies priorities for future longitudinal and implementation research.

## 5. Conclusions

Caregiver-reported HIV status disclosure among children and adolescents receiving ART remained below recommended levels in this rural South African setting. Disclosure status was associated with interconnected developmental, caregiving, and psychosocial factors, particularly child age, caregiving arrangement, caregiver readiness, and perceived need for disclosure support. Disclosure status was also associated with more favourable treatment-related indicators; however, these findings should be interpreted as cross-sectional associations rather than evidence of independent clinical effects. Overall, the findings support understanding paediatric HIV disclosure as a longitudinal caregiving process embedded within broader treatment experiences rather than a discrete event or isolated intervention. Strengthening caregiver preparation, structured counselling, and continuity of disclosure support within routine HIV services may improve implementation of age-appropriate disclosure and support transition toward sustained adolescent treatment engagement. Future longitudinal and implementation studies are needed to clarify disclosure trajectories and determine how disclosure processes influence long-term HIV outcomes.

## Figures and Tables

**Table 1:** Sociodemographic characteristics of participating children and adolescents (*n* = 87).

Participant Characteristic	*n*	%
Gender		
Female	46	52.9
Male	41	47.1
Age group		
6–9 years	16	18.4
10–13 years	47	54.0
14–19 years	24	27.6
Relationship to the child		
Biological parent	57	65.5
Legal guardian	30	34.5

**Table 2:** Association between participant characteristics and HIV status disclosure.

Participant Characteristics	Disclosed *n* (%)	Not Disclosed *n* (%)	Crude OR (95% CI)	*p*-Value
Gender of the child				0.484
Female (Reference)	19 (48.7)	27 (56.3)	Reference	
Male	20 (51.3)	21 (43.8)	1.35 (0.58–3.16)	
Age of the child				<0.001
6–9 years (Reference)	2 (5.1)	14 (29.2)	Reference	
10–13 years	16 (41.0)	31 (64.6)	3.61 (0.73–17.88)	
14–19 years	21 (53.8)	3 (6.3)	49.00 (7.24–331.60)	
Relationship to the child				<0.001
Biological parent (Reference)	16 (41.0)	41 (85.4)	Reference	
Legal guardian	23 (59.0)	7 (14.6)	8.42 (3.03–23.45)	

**Table 3:** Association between caregiver-related factors and HIV status disclosure.

Caregiver-Related Factor	Disclosed *n* (%)	Not Disclosed *n* (%)	Crude OR (95% CI)	*p*-Value
Ready to talk to the child about HIV				
Yes	38 (97.4)	16 (33.3)	76.00 (9.22–626.74)	<0.001
No (Reference)	1 (2.6)	32 (66.7)	Reference	
Belief that the child will understand HIV				
Yes	35 (89.7)	32 (66.7)	4.38 (1.30–14.73)	0.011
No (Reference)	4 (10.3)	16 (33.3)	Reference	
Knows other caregivers who have disclosed				
Yes	20 (51.3)	9 (18.8)	4.56 (1.76–11.81)	0.001
No (Reference)	19 (48.7)	39 (81.3)	Reference	
Receives emotional support				
Yes	12 (30.8)	6 (12.5)	3.11 (1.05–9.21)	0.036
No (Reference)	27 (69.2)	42 (87.5)	Reference	
Needs more training/counselling on disclosure				
Yes	10 (25.6)	44 (91.7)	0.031 (0.009–0.109)	<0.001
No (Reference)	29 (74.4)	4 (8.3)	Reference	
Afraid of how the child would react				
Yes (Reference)	34 (87.2)	48 (100.0)	Not estimable †	0.011
No	5 (12.8)	0 (0.0)		
Disclosure support preferences				
Counselling	11 (28.2)	17 (35.4)	Not applicable ‡	
Support groups	14 (35.9)	5 (10.4)		0.016
Step-by-step guidance	14 (35.9)	26 (54.2)		

† Crude odds ratio could not be reliably estimated because of quasi-complete separation resulting from a zero cell. ‡ Crude odds ratios were not calculated because this variable comprises three nominal categories without a natural reference group. The overall association with disclosure status was evaluated using the Pearson chi-square test.

**Table 4:** Association between disclosure status and treatment-related indicators.

Treatment-Related Variable	Disclosed *n* (%)	Not Disclosed *n* (%)	Crude OR (95% CI)	*p*-Value
Viral load suppression (≤1000 copies/mL)				<0.001
Yes	29 (74.4)	14 (29.2)	7.04 (2.72–18.23)	
No (Reference)	10 (25.6)	34 (70.8)	Reference	
ART adherence				<0.001
Always	34 (87.2)	22 (45.8)	6.95 (1.37–35.24)	
Sometimes	3 (7.7)	17 (35.4)	0.79 (0.11–5.65)	
Rarely (Reference)	2 (5.1)	9 (18.8)	Reference	
Medication administration				<0.001
Parent (Reference)	7 (17.9)	32 (66.7)	Reference	
Independently	32 (82.1)	16 (33.3)	9.14 (3.32–25.20)	
Adherence support systems				<0.001
Yes	36 (92.3)	25 (52.1)	11.04 (2.99–40.81)	
No (Reference)	3 (7.7)	23 (47.9)	Reference	

**Table 5:** Multivariable logistic regression model of caregiver-related factors associated with HIV status non-disclosure (*n* = 87).

Predictor	Adjusted OR (AOR)	95% CI	*p*-Value
Ready to talk to the child about HIV (Yes vs. No)	0.037	0.004–0.381	0.006
Needs more training/counselling on disclosure (Yes vs. No)	15.966	3.052–83.530	0.001
Knows other caregivers who have disclosed (Yes vs. No)	0.339	0.071–1.612	0.174
Relationship to the child (Biological parent vs. Legal guardian)	11.921	2.164–65.667	0.004

Model diagnostics: Omnibus test of model coefficients: χ^2^ = 73.439, df = 4, *p* < 0.001; Hosmer–Lemeshow goodness-of-fit test: χ^2^ = 5.097, df = 6, *p* = 0.531; Nagelkerke R^2^ = 0.763; Overall classification accuracy = 88.5%; Number of observations = 87.

## Data Availability

The datasets used are available from the corresponding author on reasonable request.
